# The Contribution of Nanomedicine in Ocular Oncology

**DOI:** 10.3390/cancers17071186

**Published:** 2025-03-31

**Authors:** Margarita Tsoplaktsoglou, Ellas Spyratou, Andreas Droulias, Maria-Eleni Zachou, Efstathios P. Efstathopoulos

**Affiliations:** 1 Medical School, National and Kapodistrian University of Athens, 11527 Athens, Greece; margtsop@yahoo.gr (M.T.); liadrou@gmail.com (A.D.); 2 Department of Applied Medical Physics, Medical School, Attikon University Hospital, National and Kapodistrian University of Athens, 11527 Athens, Greece; spyratouellas@gmail.com (E.S.); zachoumar@gmail.com (M.-E.Z.)

**Keywords:** nanomedicine, ocular oncology, retinoblastoma, uveal melanoma, drug delivery, theranostics

## Abstract

Nanomedicine is a novel and emerging field that has noted significant progress in both the fields of ophthalmology and cancer treatment. Expanding into ocular oncology, it holds the potential to overcome the limitations of conventional therapies, such as poor drug penetration due to anatomical and physiological ocular barriers and insufficient targeting, which can lead to collateral damage to healthy tissues. By reviewing a series of clinical and preclinical studies, we aim to outline the recent advancements, current trends and future perspectives in nanomedicine for ocular cancer treatment. Beyond improving the existing therapies, like chemotherapy, phototherapies and brachytherapy, nanomedicine enables multimodal applications by combining multiple treatments or integrating imaging for theranostic approaches. Additionally, it paves the way for experimental therapies, such as gene therapy, offering new possibilities for more effective and less invasive treatment strategies in ocular oncology.

## Simple Summary

This review article explores the potential use of nanomedicine in ocular oncology with the purpose of tackling issues associated with conventional treatment strategies, such as sub-optimal targeting, serious side effects and limited effectiveness. Nanomedicine holds the potential of enhancing imaging and current treatments, such as chemotherapy, photo-based therapies and radiotherapy, and even combining them, noting significant promise in the field of theranostics, multimodal applications and precision medicine. A series of preclinical and clinical studies of nanomedicines investigated in ocular oncology are presented in order to outline their advantages, current research trends and future perspectives.

## 1. Introduction

Ocular cancers, although relatively rare, often lead to devastating outcomes if not detected and treated early—not only the deterioration of vision, but poor survival due to the high percentage of metastatic cases is possible [1,2]. The current therapeutic approaches for ocular cancers, such as radiation therapy, and local or systemic chemotherapy, often come with severe side effects and limitations. These include the sub-optimal targeting of cancer cells, collateral damage to surrounding healthy tissues and inadequate penetration of ocular structures [3]. The eye, despite its small size, is one of the most complex organs of the human body, protected by a range of anatomical and physiological barriers that makes drug delivery especially challenging [4,5]. Compounding these issues, the tumor microenvironment features very particular conditions, such as an acidic pH and hypoxia, further impeding the penetration and efficacy of therapeutic agents. These challenges highlight the urgent need for innovative strategies and the improvement of cancer treatments.

Nanomedicine, the application of nanotechnology in healthcare, leverages the unique physicochemical properties of nanoscale materials to enhance drug delivery, improve pharmacokinetics and enable advanced therapeutic strategies [6]. Nanoparticles, typically ranging from 1 to 100 nm, offer a high surface-area-to-volume ratio and outstandingly customizable surfaces, allowing for large drug loads, controlled drug release as well as active targeting through ligand-based interactions [6,7]. In oncology, the tumor microenvironment allows for further precision through passive targeting due to the Enhanced Permeability and Retention (EPR) effect [8,9] and can even act as a trigger for stimuli responsive formulations [10,11], consolidating nanomedicine as a powerful tool for improving treatment efficacy while minimizing systemic toxicity.

**Figure 1:**
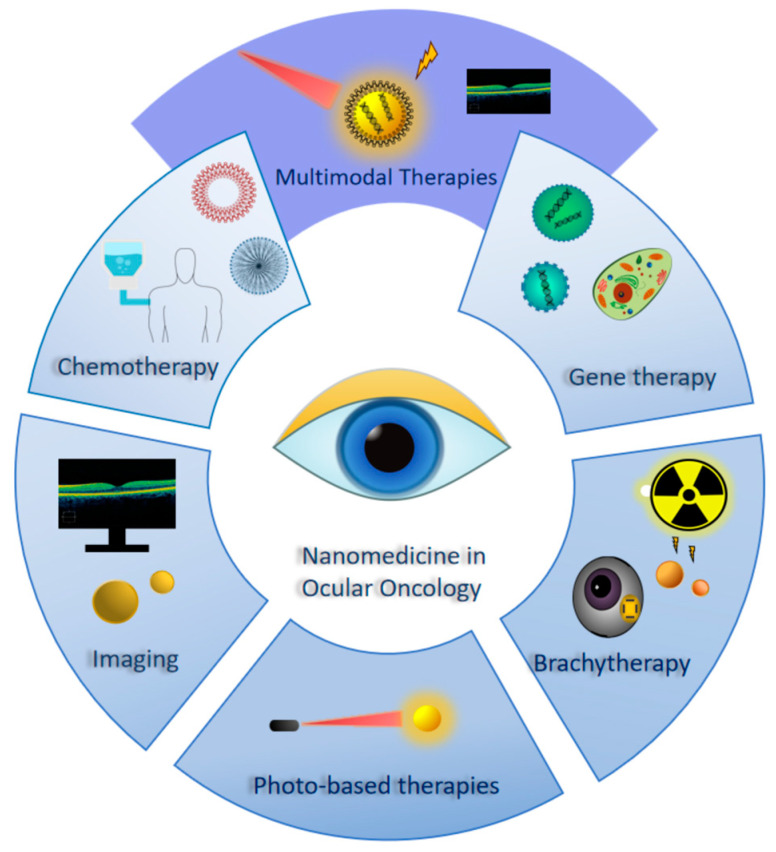
Schematic illustration of the research trends and advancements of nanomedicine in ocular oncology. Licensed under CC0 1.0 Universal.

With several nanoformulations already commercially and clinically available for ophthalmic applications [12], and several more in preclinical studies [13], the integration of nanomedicine into ocular oncology is becoming increasingly feasible. The research has explored innovative strategies to overcome drug penetration barriers, achieve targeted and sustained delivery and enhance treatment modalities, such as imaging-guided therapy, phototherapy and radiotherapy. Additionally, nanotechnology is paving the way for next-generation approaches, such as gene therapy, offering great promise to revolutionize ocular cancer treatment [14,15]. Figure 1 illustrates the fields that are trending in the nanomedicine research in regard to ocular oncology.

## 2. Nanoparticles in Chemotherapy

### 2.1. Etoposide and Carboplatin

Etoposide and carboplatin, along with Vincristine, are the three chemotherapeutic agents of choice for retinoblastoma patients, traditionally administered in a triple intravenous scheme [3]. Due to the limited penetration of systematically administered chemotherapy into the eye, along with the severe adverse effects, the need for alternative delivery systems is imperative.

Over a decade ago, Mitra et al. first demonstrated the genetic impact of etoposide-loaded poly(lactic-co-glycolic acid) (PLGA) NPs, showing an upregulation of apoptotic gene activity in Y-79 cancer cells when compared to native etoposide, achieving about 100-times-greater anti-proliferative activity (IC50 = 0.002 μg/mL versus IC50 = 0.2 μg/mL). This finding highlighted the potential of nanoparticulate delivery systems to not only improve drug delivery but also to influence cellular mechanisms at a genetic level, paving the way for innovative approaches in retinoblastoma therapy through toxicogenomics [16].

Further advancements in this field were made by Godse et al., who formulated PLGA NPs of etoposide specifically for intravitreal injection. They modified the surface of these NPs with chitosan, a mucoadhesive polymer known for its ability to stabilize drug-loaded PLGA complexes and promote sustained drug release at the target site [17]. Chitosan’s free amino groups also allow for the conjugation of targeting ligands, such as galactose, which was selected in this case to target the lectins overexpressed in retinoblastoma cells. The resulting galactose–chitosan etoposide PLGA NPs (GC-ENPs) exhibited a sustained drug release pattern and significantly enhanced cellular uptake by Y-79 retinoblastoma cells that overexpressed lectins, compared to non-conjugated PLGA–etoposide NPs. Notably, the cytotoxicity and apoptosis rates in Y-79 cells treated with GC-ENP were greater than those observed with plain etoposide formulations (cell viability of 57.32% with free etoposide versus 53.55% with GC-ENPs, *p* < 0.0001) [17].

Other types of NPs that have been used for the ocular delivery of etoposide include Solid Lipid Nanoparticles (SLNs). In a study by Ahmad et al., etoposide-loaded SLNs were optimized using a Design of Experiments (DoE) approach to enhance drug bioavailability and retention in ocular tissues. The etoposide-loaded SLNs were able to sustain a therapeutic concentration in the vitreous body for 7 days after a single intravitreal injection, with enhanced deposition in areas like the cornea, conjunctiva and retina [18].

As far as carboplatin is concerned, Kang et al. crafted PAMAM dendritic NPs, which they loaded with carboplatin and administered subconjunctivally in mice. They found that the same dose of carboplatin had better results in terms of tumor shrinkage as a nanoparticulate formulation than the conventional carboplatin in an aqueous solution (mean tumor burden of 198,392 ± 117,373 square pixels for eyes treated with nanoparticle carboplatin at 10 mg/mL versus 724,956 ± 186,433 square pixels for eyes treated with carboplatin in an aqueous solution, *p* < 0.01). They were also able to administer higher doses of the drug in its NP form than is possible with plain carboplatin due to its instability. With the higher dose, they had comparable results to low-dose NP carboplatin in the treated eye (mean tumor burden of 82,064 ± 85,503 square pixels with nanoparticle carboplatin; 37.5 mg/mL), but they also observed tumor regression in the fellow eye, which suggests a systematic effect [19].

The mesoporous silica NP (MSNP) is another type of NP that has been used for the delivery of carboplatin. Specifically, Qu et al. conjugated MSNPs with an Epithelial Cell Adhesion Molecule (EpCAM) antibody, as EpCAM is a transmembrane glycoprotein that is often overexpressed in rapidly proliferating epithelial tumor cells. The addition of the EpCAM antibody offered a remarkable advantage to carboplatin–MSNPs compared to MSNPs without it, in terms of targeting retinoblastoma cells and enhancing their cellular uptake, resulting in better efficacy of the drug [20].

Another strategy to improve targeted delivery is decorating NPs with proteins that belong in the transferrin group, leveraging the overexpression of transferrin receptors in tumor cells. Yet, Ahmad et al. followed a different approach and directly used the proteins apotransferrin and lactoferrin as carriers for carboplatin. The resulting apo-nano-carbo and lacto-nano-carbo exhibited high specificity and efficient cellular uptake by Y-79 retinoblastoma cells during in vitro testing. They also demonstrated a pH-dependent release of carboplatin, responding to the acidic environment typical of tumor cells, and exhibited a prolonged residence time compared to soluble carboplatin [21]. Taking it a step further, Narayana et al. fabricated lactoferrin protein NPs, which they loaded with both etoposide and carboplatin. When tested in vitro on cancer stem cells in the Y-79 retinoblastoma cell line, the NPs exhibited a significantly higher cellular uptake compared to non-cancer cells, demonstrating remarkable specificity, and with the added benefit of dual chemotherapy, notable cytotoxicity [22].

Following a similar approach, Shinde et al. also developed PLGA NPs that combined carboplatin and etoposide (CE-MIP). The in vitro cytotoxicity studies revealed that CE-MIP outperformed the plain drug mixture against Y-97 cells. Subsequent testing on an orthotopic mouse model via subconjunctival administration showed that CE-MIP elicited double the apoptotic effect compared to the control groups (15.7% versus 7.9%) [23].

These studies underscore the potential of NP-based drug delivery systems for active targeting, sustained release as well as environment-triggered release, improved bioavailability and the co-administration of chemotherapeutic agents. Furthermore, they provide alternative routes of administration, i.e., subconjunctivally/intravitreally, to overcome the disadvantages of intravenous chemotherapy.

### 2.2. Vincristine

Vincristine is a powerful chemotherapeutic agent that has been used in both retinoblastoma and uveal melanoma, amongst other non-ocular cancers. Notably, Marqibo^®^; Acrotech Biopharma Inc., East Windsor, NJ, USA, a liposomal formulation of Vincristine sulfate, was first approved in 2012 for the treatment of Philadelphia chromosome-negative acute lymphoblastic leukemia (ALL). Its potential but also toxicity have been explored for metastatic melanoma of uveal, cutaneous or mucosal origin with a good safety profile [24]. Subsequently, a higher dose was tested in a phase 2 trial for patients with metastatic uveal melanoma [25]. Liposomal Vincristine was later investigated in two phase 3 trials led by the Children’s Oncology Group, as part of an adjuvant chemotherapy regimen for pediatric patients with intraocular retinoblastoma [26,27].

Further research is being conducted into Vincristine delivery through the development of NP-based systems. For example, Vincristine-loaded Pluronic F127 polymer-coated magnetic NPs were synthesized by Sadri et al. [28]. This group used two different ligands simultaneously, folic acid (FA) and transferrin, in order to increase selectivity and drug uptake. The VCR-loaded NPs demonstrated significantly lower IC50s against Y79 cells compared to healthy ARPE19 cells, which suggests higher selectivity and better drug penetration into Y79 cells.

In addition to these advantages, the magnetic NPs exhibited superparamagnetic properties, allowing them to absorb heat effectively and to even generate ROS. Under alternating magnetic field (AMF) irradiation, the NPs heated Y79 cells more efficiently, demonstrating that the combination of hyperthermia and chemotherapy was far more effective than either treatment alone, highlighting the synergistic benefits of this dual approach [28].

### 2.3. Doxorubicin

Doxorubicin is a widely used chemotherapy drug with applications for different types of cancer. It has thus been a point of interest in the research to be implemented into targeted delivery systems. In fact, Doxil is the first chemotherapeutic drug and one of the first nanoformulations overall to have been made commercially available. This unilamellar PEGylated liposomal formulation of doxorubicin was first introduced in 1995, and is approved for the treatment of ovarian cancer, AIDS-related Kaposi’s sarcoma and multiple myeloma. Since then, more nanoformulations of doxorubicin have emerged. Lipo Dox^®^ is the second generation of PEGylated liposomal doxorubicin with a longer half-life and better stability than its predecessor. A non-PEGylated version of liposomal doxorubicin, Myocet, has also been made available since 2000 for the treatment of metastatic breast cancer. Myocet’s performance has been equal to that of traditional doxorubicin, but without the cardiotoxic effects associated with it.

The continuous interest in refining doxorubicin formulations is exemplified by innovations, such as PLGA nanocarriers, which were developed by Park et al. These nanocarriers were further enhanced with gold half-shells, forming PLGA-Au NPs loaded with doxorubicin, which could be used in phototherapy. These NPs, upon NIR irradiation, could simultaneously deposit heat and release the drug at the tumor site, drastically improving the cytotoxicity for cancer cells while sparing healthy tissues [29].

Similar principles have been applied in the treatment of retinoblastoma by different research groups. Boddu et al. achieved a sustained release of doxorubicin over two weeks by incorporating folate-receptor-targeted PLGA-PEG-FOL micelles in a thermosensitive PLGA-PEG-PLGA gel. This sustained release is crucial for maximizing drug exposure to the tumor cells while minimizing the need for frequent administration. The study also demonstrated the targeted delivery capability of these micelles, revealing a fourfold increase in doxorubicin uptake by Y-79 retinoblastoma cells compared to free doxorubicin. This enhanced uptake was attributed to the presence of folate receptors on the surface of Y-79 cells, which readily bind to the folate conjugated on the micelles [30].

Parveen et al. also capitalized on the overexpression of folate receptors in Y-79 cells and created folate-decorated chitosan NPs. In vitro studies with Y-79 cells demonstrated the superior cytotoxic effect of the folate-conjugated doxorubicin-loaded chitosan NPs compared to free doxorubicin or unconjugated NPs, as they had an advantage in cellular internalization via receptor-mediated endocytosis. This targeted approach not only improves drug delivery to the tumor site but also has the potential to reduce the dosage required, thereby minimizing off-target effects and improving the overall safety profile of doxorubicin treatment [31].

Another revolutionary approach presented by Gao et al. is pH-responsive nanoceria, to specifically address the challenge of drug delivery to the tumor microenvironment. As has been discussed previously, nanoceria act as antioxidants in physiological pH environments. However, the acidic tumor environment leads to changes in their electronic structure causing them to act as oxidases and to generate ROS, thus exhibiting cytotoxic activity [10]. Additionally, the nanoceria were coated with glycol chitosan and conjugated with doxorubicin and AMD11070, a CXCR4 antagonist. The chemokine receptor CXCR4 has been found to be overexpressed in various cancers, and the group showed that retinoblastoma is amongst those. Interactions between CXCR4 and its ligand, CXCL12, support tumor growth by enhancing angiogenesis, immune evasion and resistance to therapies, as well as tumor metastasis. AMD11070 not only disrupts the CXCR4–CXCL12 axis but also facilitates tumor-specific targeting. This triple approach of pH-activated cytotoxic nanoceria, the CXCR4 antagonist and the traditional chemotherapeutic agent, doxorubicin, demonstrated a synergistic effectiveness against retinoblastoma, both in vitro and in vivo [32].

### 2.4. Other Chemotherapeutic Agents

Melphalan is one of the novel chemotherapeutic agents that has been applied in the treatment of retinoblastoma, injected either intravitreally or intra-arterially. In the past few years, it has been involved in NP-mediated delivery studies. Sims et al. created melphalan-loaded PLGA NPs, which they compared to free melphalan in vitro against retinoblastoma. They also experimented with modifying the surface of the NPs with biotinylated peptides, namely PEG, TET1 and MPG, to improve stability and enhance targeted delivery. All modified NPs performed better than unmodified ones, with the latter being the most effective and achieving the highest internalization by Y-79 cells [33].

Even more ambitious projects with melphalan have been overtaken. To overcome the risks related to injections altogether, Moheni et al. developed chitosan–alginate melphalan NPs for topical administration. However, since sodium alginate is anionic and hydrophilic, its ability to diffuse through the cornea—which typically favors hydrophobic and cationic or neutral compounds—poses a significant challenge. To address this, the NPs were modified with lauric acid, which not only enhances the formulation’s hydrophobicity, but also possesses mucoadhesive properties. Ex vivo and in vivo testing in bovine eyes, along with imaging, showed that the modified chitosan–alginate NPs successfully permeated the cornea and delivered melphalan to the vitreous cavity, potentially revolutionizing the treatment of retinoblastoma [34].

Topotecan is another chemotherapeutic drug, approved for various cancers, such as small cell lung carcinoma and ovarian and cervical cancers, and has also been investigated for retinoblastoma [35]. However, its potential use has suffered due to low bioavailability, side effects and pH-dependent hydrolysis, which converts it from its active lactone form to a biologically inactive carboxylate species under physiological conditions. Delrish et al. aimed to find a more viable option by incorporating topotecan into chitosan NPs. In vitro tests on human retinoblastoma cells exhibited better rates of cytotoxicity compared to free topotecan, and those results were further demonstrated by in vivo testing in a rat model (IC50 53.17 nM for topotecan with thiolated chitosan NPs compared to 138.30 nM for free topotecan, *p* = 0.01) [36]. The same group further experimented with thiolated and methylated chitosan carboxymethyl dextran NPs (CMD-TCs-NPs and CMD-TMC-NPs) labeled with Cy5 to determine which NPs had the optimal distribution in the virtuous post-intravitreal injection in rats with retinoblastoma. They determined that the former had better diffusion capabilities due to a lower positive surface charge, and the thiol groups facilitated their interaction with the Y79 cells, potentially improving drug delivery and efficacy [37].

Following their experiments with carboplatin-loaded MSNPs, Qu et al. also developed topotecan-loaded MSNPs, which they decorated with FA. Their study showed a remarkable internalization of the decorated MSNPs through receptor-mediated endocytosis, resulting in significantly higher levels of cytotoxicity in retinoblastoma cells and shrinkage of the tumor in vivo, compared to both free topotecan and unconjugated NPs [38].

Dasatinib is a small-molecule tyrosine kinase inhibitor that has received approval and is used for the treatment of imatinib-resistant chronic myelogenous leukemia (CML) and Philadelphia chromosome-positive (Ph+) acute lymphoblastic leukemia (ALL). As a dual inhibitor of both Src and ABL kinases, the deregulation of which plays a key role in oncogenetic processes, it has been studied and demonstrated anti-tumor effects in a plethora of human cancers, such as triple-negative breast, gastric and pancreatic cancers and has even progressed to phase I clinical trials against metastatic cutaneous melanoma [39].

Given its broad spectrum of activity, the potential application of dasatinib in uveal melanoma has also been explored. In preclinical trials, dasatinib was shown to inhibit the growth of primary uveal melanoma cells by disrupting the ERK signaling pathway, a MAPK pathway that is responsible for malignant growth and whose activation depends on the Src kinase. That was further supported by the observation that uveal melanoma cells with higher Src activity and MAPK activation were more susceptible to growth inhibition by dasatinib [40,41]. It is worth noting that dasatinib-loaded polymeric micelles have been constructed for intravitreal use and evaluated for safety both in vivo and in vitro [42]. Although these experiments focused on proliferative vitreoretinopathy, they lay the groundwork for future applications in treating other ocular diseases, including uveal melanoma.

Other, less conventional agents, like curcumin, have also been explored in nanomedicine for treating ocular cancers. Curcumin has been shown to sensitize drug-resistant tumors by reversing multidrug resistance (MDR) through acting on several MDR pathways, such as the PI3K/Akt/NF-κB, JAK/STAT, MAPK and p53 pathways [43,44,45]. In the Y-79 cell line specifically, it was shown by Thiyagarajan et al. that it modulates the lung-resistance protein (LRP), which contributes to a particularly challenging form of drug resistance in retinoblastoma. These findings, along with the instability and poor bioavailability of curcumin, inspired Das et al. to incorporate it in PLGA NPs, which they decorated with folate. They also added nutlin-3a in the PLGA NPs, an anticancer drug whose efficiency they hypothesized would be enhanced by the presence of curcumin. Indeed, their in vitro experiments against Y-79 cells showed that curcumin inhibits the MPD genes and proteins—MRP-1 and LRP. It also acts synergistically with nutlin-3a to improve cytotoxicity, cell cycle arrest, loss of mitochondrial membrane potential (MMP) and induction of apoptosis. In fact, folate/nutlin-3a/curcumin NPs performed superiorly compared to single-drug treatments, unconjugated dual-drug-loaded NPs and native drugs in combination [46].

Curcumin’s potential has also been explored in other ocular cancers, such as uveal melanoma. Xie et al. developed an in situ hydrogel that uses hyaluronic acid and collagen, which are basic components of the vitreous body. Curcumin-loaded NPs were embedded in the hydrogel, which could pass from its liquid form in room temperature to its gel form at 37 °C within two minutes. This delivery system proved to be biocompatible and slow degrading, allowing a sustainable release. Most importantly, the gel that contained curcumin-loaded NPs resulted in significantly lower cell viability of uveal melanoma cells compared to the gel containing the same amount of curcumin without the NP carriers. The group suggests this may be due to the instability of curcumin in the aqueous environment and proves the necessity of NPs for more stable, sustainable and effective drug delivery [47].

Latorre et al. employed albumin-stabilized gold nanoclusters loaded with AZD8055, an mTOR kinase inhibitor that exerts its oncolytic effect via the PI3K/AKT/mTOR pathway for the treatment of uveal melanoma. While free AZD8055 is impossible to administer intravenously due to its hydrophilicity, the albumin-based NPs aided its systemic administration in mice and its reach to uveal melanoma (Mel202) cells. They also demonstrated excellent specificity by sparing healthy keratinocytes as opposed to unconjugated AZD8055 [48].

Pentacyclic triterpenes have also demonstrated their anti-tumor potential, and with the help of nanomedicine, they can achieve desirable solubility, stability and bioavailability to be considered for cancer treatment [49,50]. Silva et al. developed PLGA NPs loaded with Oleanolic (OA) or its isomer, ursolic acid (UA), which they tested against three different human cancer cell lines, among which were Y-79 cells. For the retinoblastoma cell line in particular, both types of PLGA NPs exhibited significant cytotoxicity, accentuating their potential in retinoblastoma treatment [51].

Lastly, Methotrexate, which is the main chemotherapeutic drug for intraocular lymphoma, has been successfully conjugated with dendrimers by two different research groups. Both groups used PAMAM-based formulations functionalized with FA, and demonstrated that the slower diffusion rate of the nanosystem compared to the free drug resulted in controlled release with better therapeutic results, as well as reduced toxicity [52,53].

## 3. Nanoparticles in Ocular Imaging

There are two approaches through which NPs can contribute to ocular imaging. One approach is to conjugate conventional imaging enhancers with NPs, in order to improve their stability and targeting. For example, liquid pentafluropentane (PFP) has been conjugated with both organic and inorganic NPs to enhance ultrasound (US) and photoacoustic imaging (PAI), as mentioned in this article. The second approach is to use NPs themselves as enhancers. Inorganic nanoparticles, especially gold nanoparticles (AuNPs), have been extensively studied and proven themselves as excellent contrast agents for imaging modalities, such as Optical Coherence Tomography (OCT) and PAI, due to their tunable scattering and absorbing properties that they owe to the LSPR effect. OCT functions by detecting the backscattering of light from the tissues, typically using NIR light, to create an interference pattern and provide cross-sectional images. PAI is a hybrid imaging technique that depends on the photoacoustic effect and combines light (laser) and ultrasound to visualize tissues. It works by using laser pulses to heat tissues that then, due to thermoelastic expansion, generate pressure waves detectable as ultrasound that ultimately generate the PAI image. PAI also remains largely unaffected by scattered lighting, allowing deeper tissue imaging than OCT. AuNPs can both amplify the backscattered light measured by OCT and convert light energy into heat and acoustic waves that PAI detects [54,55].

Over a decade ago, Prabhulkar et al. used gold nanorods to detect squamous cell carcinoma in conjunctival cells. They functionalized the nanorods with antibodies specific for glucose transporter-1 (GLUT-1), a protein overexpressed in ocular surface squamous neoplasia (OSSN) to visualize neoplastic cells on the OCT. Their study faced a few notable limitations, including the low specificity of GLUT-1 antibodies, which also bound to erythrocytes, and the need for a “threshold” concentration of Au nanorods in the tissue for detection via OCT. This threshold was only reached by more advanced OSSN lesions due to higher GLUT-1 overexpression. Yet, they successfully showed that Au nanorods can be used as molecular markers for OSSN on the OCT [56].

Raveendran et al. were the first to use AuNPs for ocular PAI. Their nanocages, which can be synthesized remarkably fast, were demonstrated to emit strong PA signals when excited with a nanosecond-pulsed laser and produced high-contrast PA and US images. In fact, ex vivo experiments on porcine eyes demonstrated approximately 50% stronger PA signals in the presence of nanocages, which the team postulated could be leveraged to more precisely visualize the size, depth and location of uveal melanoma [57].

Warther et al. crafted mannose-functionalized fluorescently labeled MSNPs designed to target retinoblastoma (Rb) cells. This strategy was based on their previous work demonstrating that both mannose and galactose can facilitate the uptake of NPs by Rb cells through carbohydrate receptor-mediated endocytosis, a process driven by the overexpression of these receptors on Rb cells. The researchers focused on synthesizing MSNPs with a diameter of approximately 25 nm, small enough to potentially bypass the blood–retinal barrier. To address the challenge of aggregation commonly encountered with small MSNPs, PEG chains were incorporated onto the NP surface. The successful uptake of the fluorescently labeled MSNPs by the target cells was confirmed by confocal microscopy allowing the detection of retinoblastoma in vitro [58].

While the aforementioned studies primarily focused on imaging in ocular cancers, most NP-mediated imaging is actually conducted in combination with therapeutic interventions. The following sections explore several multimodal applications of NPs, highlighting their dual role in both diagnosis and treatment.

## 4. Nanoparticles in Photo-Based Therapies

NPs have gained popularity as agents for controlled and targeted heat deposition, thus causing minimal damage to healthy tissue. Their optical properties in combination with their specific binding at tumor cells have given rise to promising types of therapies that rely on hyperthermia or photoactivation. In particular, Photothermal Therapy (PTT) and Photodynamic Therapy (PDT) were initially developed without NPs, relying on direct tissue heating or conventional photosensitizers, respectively, but the introduction of nanotechnology has greatly enhanced them.

**Figure 2:**
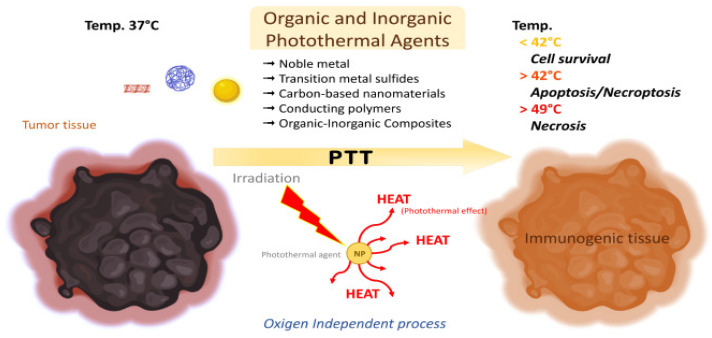
The cytotoxic effects of PTT through hyperthermia and commonly employed photothermal agents. Reprinted/adapted with permission from [62], 19 February 2025, Elsevier.

PTT uses light-sensitive heating NPs which, after being delivered to the tumor site, are activated by a particular wavelength, usually in the near-infrared (NIR) spectrum. Different types of NPs, such as AuNPs, quantum dots and graphene oxide, have been employed for their optical properties, but conventional NIR agents, such as indocyanine green (ICG), can also be used in conjugation with other types of NPs (Figure 2). Nanomaterial-mediated PTT has been a subject of investigation for several types of tumors, such as oral epithelial [59], colon [60] and lung carcinoma [61], and due to it being effective and minimally invasive, it is only logical that it would be studied for ocular cancer treatment as well [62].

**Figure 3:**
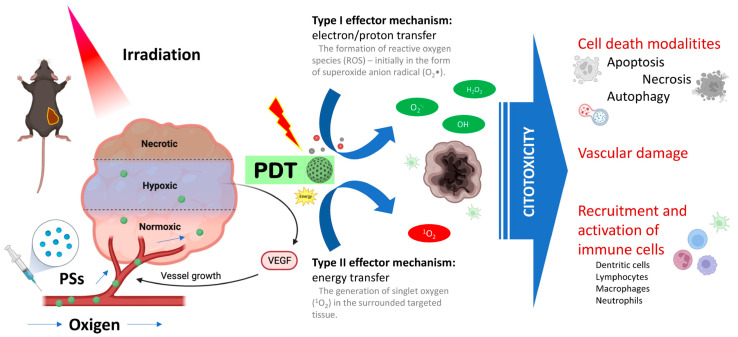
The two effector mechanisms of PDT. Type I: the photosensitizer in its excited state transfers photons/electrons to the surrounding biomolecules, resulting in the formation of superoxide anion radicals (O_2_•) and then ROS inside cells. Type II: the photosensitizer transfers its energy directly to molecular oxygen (O_2_) to generate a singlet oxygen (^1^O_2_) that has powerful reactive and oxidizing potential. Reprinted/adapted with permission from [62], 19 February 2025, Elsevier. red circle; singlet oxygen (^1^O_2_); gray circle; cancer cells; OH: hydroxyl radical.

PDT uses photosensitizers that, upon light irradiation, can transfer absorbed photon energy or excited electrons to surrounding oxygen to generate reactive oxygen species (ROS), thus damaging specific target cells. Due to their rapid growth, abnormal metabolism and poor blood supply, tumor cells exhibit higher levels of ROS species than normal cells, thus PDT capitalizes on their higher redox potential (Figure 3).

Similarly to imaging enhancers, NPs can act as photothermal agents or photosensitizers, or they can be used as carriers for conventional agents used in PTT and PDT. In either case, nanotechnology addresses some important limitations of traditional agents by offering more stable solutions that do not form aggregates and do not degrade as fast, resulting in more effective and prolonged action.

### 4.1. Hyperthermia

Tumor cells are known to be more susceptible to heat than healthy cells due to hypoxic and acidic conditions that occur from their poor vasculature, and thus heat-induced apoptosis has been explored as an anticancer strategy [63]. Nevertheless, achieving further precision is crucial to minimize damage to surrounding healthy tissues. The advantage of using nanotechnology for improved selectivity and less collateral damage has been well demonstrated in the research.

Demirci et al. crafted dextran-coated iron oxide NPs (DCIONs) to induce magnetic hyperthermia. When an external alternating magnetic field is applied, magnetic NPs oscillate, generating heat through three mechanisms: Néel relaxation, Brownian relaxation and hysteresis loses. In addition, they can produce ROS, resulting in further DNA damage and protein oxidation. The group treated both Y-79 and ARPE-19 cell lines, the latter being healthy RPE cells, to magnetic hyperthermia and determined that in the presence of DCIONS, cell apoptosis was selectively triggered in retinoblastoma cells depending both on the dose and time following irradiation, with minimal cytotoxicity to healthy cells [64].

Ultrasonic hyperthermia is another heat-depositing treatment that has been implemented in retinoblastoma, where high-frequency sound waves (ultrasound) are used to generate localized heat in a tissue. Moradi et al. hypothesized that AuNPs can enhance the efficacy of ultrasonic hyperthermia against retinoblastoma cells. Indeed, their in vitro experiments showed that cell apoptosis of 50% was achieved in just 4.5 min of radiation for Y97 cells in the presence of AuNPs instead of the 9 min that were required with ultrasonic radiation alone. In addition, the cytotoxic effect was proportional to the concentration of AuNPs, proving that Y97 cells are more susceptible to hyperthermia in the presence of AuNPs in a dose-dependent manner [65].

Laser-induced hyperthermia is another concept that has been explored and successfully implemented for cancer cell apoptosis, especially with the involvement of AuNPs. With a similar concept to SLT (Selective Laser Trabeculoplasty) that is routinely used in clinical practice for the treatment of open angle glaucoma by targeting the pigmented cells of the trabecular meshwork, Pitsillides et al. developed a method that used short laser pulses to ensure that heat does not have time to flow away and is confined at the target, a phenomenon known as thermal confinement. Their technique relied solely on light absorption, specifically the interaction of light with NPs. The NPs of choice were AuNPs that were conjugated to different antibodies for targeted delivery. The group experimented with laser parameters, such as wavelength and duration, as well as NP size and concluded that AuNPs could render the plasma membranes of target cells transiently permeable and inactivate their proteins, resulting in their selective death upon treatment with the nanolaser [66].

Katchinskiy et al. explored a similar technique for the treatment of retinoblastoma. Specifically, they used PEGylated gold nanorods coated with EpCAM antibodies for targeted delivery. They also utilized femtosecond lasers, which have an even shorter pulse duration than nanolasers and can achieve higher peak power, leading to very high temperatures for the gold nanorods, beyond the melting point of bulk gold. The nanorods can then exert their thermal properties to the environment, which are confined to the cell they are in, without affecting neighboring cells, leading to the targeted apoptosis of tumor cells. Their choice of wavelength for the laser pulses at 800 nm was also key to the optimal effect of the laser, because it falls within the “tissue transparency window” of the eye. This means that light at this wavelength can pass through the cornea, lens and other eye structures with minimal absorption before reaching the retina [67].

The following year, Darviot et al. used a 527 nm nanosecond laser, which is conventionally used for vitreolysis in higher fluences. For their experiment, they used AuNP and AuAgNPs with lower fluences to irradiate Y79 cell cultures in a vitreous phantom model made of hyaluronan to reproduce the viscosity of the vitreous body. AuNP and AuAgNPs achieved similar results, so they settled on AuNPs as they are considered less toxic. They proved that the presence of AuNPs can lower the fluence required for apoptosis as they produced a cellular death rate of 80% with a fluence value of 20 J cm^−2^ cell clusters. Another concern is that Y79 cells tend to form aggregates that are harder to reach by irradiation alone, but AuNPs succeeded in diffusing within the clusters even in a viscous medium, achieving cell death despite the aggregates [68].

### 4.2. Light-Activated NPs/Photosensitizers

For the treatment of retinoblastoma, there are instances of NP-based photosensitizers designed for PDT from over a decade ago. For example, Makky et al. created liposomes to carry glycodendrimeric porphyrins, which are commonly used for their photodynamic activity. They experimented with porphyrin compounds both with and without mannose residues. The liposomes with the porphyrin compounds were tested in an artificial Rb cell membrane model, and it was concluded that mannosylated porphyrins could bind more effectively to Rb cells and be potentially used in PDT [69].

Contrary to being used as carriers for conventional photosensitizers, some NPs have been explored as photosensitizers themselves, such as titanium dioxide (TiO_2_) NPs, which have exhibited notable photocatalytic activity. Balachadran et al. tested both pure TiO_2_ and cerium (Ce)-doped TiO_2_ NPs against Y-79 cells for their photodynamic capacity. It was determined that Ce–TiO_2_ was significantly more cytotoxic against cancer cells upon UV radiation exposure, which was attributed to the light-responsive capability and strong redox properties of cerium [70].

For the treatment of uveal melanoma, Normand et al. created light-sensitive NPs, called vectosomes, formed by complexing the C-terminal half of the VP22 protein with antisense oligonucleotides (ODNs). They used the antisense c-raf ODN to target and inhibit the expression of the c-raf kinase gene, which is crucial to cancer cell proliferation and the prevention of cell death. The vectosomes were tested both in vitro and in vivo, in a mice model, and were found to successfully reach the retina when intravitreally injected and were internalized by both the OCM-1 (uveal melanoma) and ARPE-19 (normal RPE) cells lines, where they remained stable, unless illuminated. Upon transscleral illumination with either cold white light or an Argon laser, the ODNs were released and quickly reached the nucleus, inhibiting OCM-1 cell proliferation by up to 60% compared to untreated or unilluminated groups [71].

Perhaps the most ambitious and promising treatment currently investigated for the treatment of uveal melanoma is Belzupacap sarotalocan (AU-011), a light-activated NP. It consists of a human papillomavirus (HPV) virus-like particle (VPL) conjugated with IRDye^®^ 700, a phthalocyanine photosensitizer that responds to NIR irradiation. The VPL can deliver over 40 times the amount of dye molecules compared to the alternative, antibody–drug conjugates [72]. It is also designed to specifically target heparan sulfate proteoglycans (HSPGs), which are often overexpressed and mutated in tumor cells [73]. In vitro studies have demonstrated that, when activated by NIR light, AU-011 can induce immunogenic cell death in uveal melanoma cell lines [74]. Subsequent in vivo studies on rabbits explored both intravitreal and suprachoroidal administration to determine optimal drug distribution and penetration into the choroid [75]. Suprachoroidal administration proved superior and advanced to a phase 2 clinical trial of AU-011 for patients with primary indeterminate lesions and small choroidal melanoma (NCT04417530) [76].

Last but not least, another promising formulation that capitalizes on the benefits of the NP-assisted delivery of photosensitizers is Visudyne^®^; Cheplapharm Arzneimittel GmbH, Greifswald, Germany. Visudyne^®^ is a lipid-based nanoformulation of the photosensitizer verteporfin and the only commercially available formulation of verteporfin. It has been clinically approved for PDT therapy in AMD and choroidal neovascularization, and it is now being explored for PDT therapy in uveal melanoma. Multiple case series with verteporfin-mediated PDT have been conducted for the treatment of small choroidal melanoma, including three studies of 45 patients in total, which showed tumor regression for most patients, stable or improved visual acuity and the depletion of subretinal fluid, which is an indicator of abnormal leaking vessels. One of the most recent case series of 12 patients showed a 67% rate of complete tumor regression following three sessions of PDT after an average of 5 years. However, another study with a 5-year follow-up time and 26 patients suggests the possibility of recurrent malignancy and proposes other therapies be prioritized [77]. Nonetheless, all four studies demonstrated that PDT with Visudyne was well tolerated and without significant complications [78,79,80]. It is also worth noting that another clinical study was conducted by Sun Yat-sen University to evaluate the safety and effectiveness of Visudyne-mediated PDT in retinoblastoma, though the results are yet to be made available (NCT04429139) [81].

## 5. Multimodal Applications

A revolutionary aspect of using NPs for photothermal therapies is that they can be combined with in vivo imaging, targeted chemotherapy as well as with each other. While some examples of imaging or photo-based therapies have been mentioned separately, most studies capitalize on the multimodal capabilities of NPs. The combination of multiple treatments has several benefits, such as an additive or synergistic effect, the potential to use lower, safer doses of radiation or chemotherapeutic drugs to achieve therapeutic results and less frequent interventions, while theranostics allow for close monitoring, control and precision.

### 5.1. PTT in Retinoblastoma

Lipid-based NPs are at the forefront of the current research for combining the PTT of retinoblastoma with other modalities, due to their versatility and ability to integrate different types of molecules. Liu et al. utilized liposomes to encapsulate and stabilize ICG, a NIR agent that is FDA approved for PTT but whose use often suffers due to issues of aggregation, instability and fast clearance. In vitro and in vivo experiments on mice with retinoblastoma showed that its conjugation to liposomes not only prevents its premature clearance through systemic circulation, but also improves delivery to the target site with better tissue penetration. As a result, the ICG-liposomes greatly enhanced fluorescence and PA imaging in vivo and boosted the efficacy of PTT, advancing the development of image-guided phototherapy [82].

**Figure 4:**
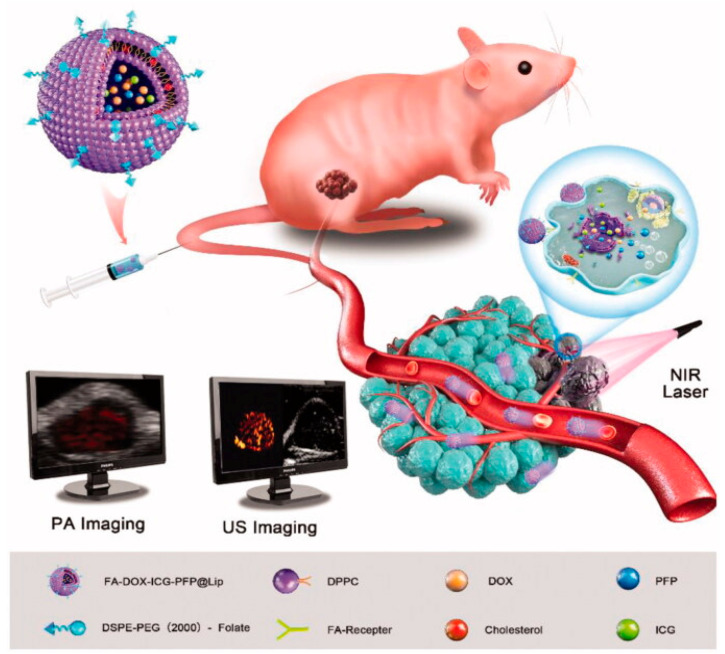
FA-DOX-ICG-PFP@Lip used for the synergistic photothermal and chemical therapy of retinoblastoma under the guidance of dual-modal PA/US imaging. Reprinted/adapted with permission from [83], 19 February, Taylor & Francis. Licensed under a Creative Commons Attribution 4.0 International License.

In the same year, Li et al. also developed ICG-liposomes, additionally incorporating doxorubicin for combined chemotherapy, along with liquid PFP to further enhance imaging. PFP is a fluorocarbon that is liquid at room temperature but has a relatively low boiling point, vaporizing to form microbubbles that improve the clarity of US imaging with slight increases in temperature. Furthermore, they decorated the nanoplatform with FA to specifically target the folate receptors of Rb cells. The resulting FA-DOX-ICG-PFP@Lip could be monitored in real time with PAI, and upon irradiation with an 808 nm NIR laser in vitro, they could quickly produce high temperatures that induced the apoptosis of Y-79 cells. Simultaneously, they would transition from liquid to gas, serving as highly effective contrast agents for US imaging, while a burst of doxorubicin release would also occur. Subsequently, the group proceeded to in vivo experiments in Y-79 tumor-bearing mice to assess the distribution and biocompatibility of the NPs, while monitoring them with dual PA/US imaging (Figure 4). They found their size allowed them to be internalized by tumor cells through the EPR effect in addition to folate receptor-mediated endocytosis. Healthy cells and the hematopoietic cell lines (WBC, RBC, platelets) remained largely unaffected, deeming the liposomal formulation safe in this regard, as opposed to conventional chemotherapy. Finally, the cancer killing efficacy of the FA-DOX-ICG-PFP@Lip was evaluated and proved to be higher than all the other treatment groups, such as free doxorubicin alone, irradiation alone, non-targeted DOX-ICG-PFP@Lip or FA-DOX-ICG-PFP@Lip without a laser. This is a pioneering study in retinoblastoma that demonstrates the multi-functionality of NPs in theranostics, as well as the synergistic effect of multiple therapeutic approaches as opposed to individual treatments [83].

Lipid-based NPs were also explored by Wang et al. for combined chemotherapy and PTT of retinoblastoma. More specifically, the group encapsulated melphalan and black phosphorus quantum dots (BPQDs) in lipid NPs. LNP@Me&BP exhibited good photothermal properties due to the BPQDs both in vitro and in vivo, and enhanced the cytotoxic effect of melphalan on Rb cells. Surprisingly, the in vivo experiments on mice revealed that BPQDs alone had a slightly stronger inhibitory effect on tumor cell proliferation and greater reduction in the ki67 level than LNP@Me&BP, which the team attributes to the greater concentration of BPQDs than what was used in the lipid nanoformulation. Nonetheless, high doses of BPQDs are associated with side effects, such as nephrotoxicity, inciting the necessity of lower doses. The LNP@Me&BP system accomplished this by combining melphalan with a lower concentration of BPQDs resulting in comparable tumor-killing efficacy and with a better safety profile [84].

Other types of organic NPs have also been investigated for the same purpose. Mudigunda et al. designed polymeric NPs (PNPs) from PLGA and PCL, which they conjugated with a NIR dye, IR820 (IR), and the chemotherapeutic drug Palbociclib (PCB), a CDK4/CDK6 inhibitor. The PCB/IR PNPs were excellent photothermal agents as well as contrast agents for PA imaging, and the synergistic effect of PTT and chemotherapy compared to individual therapies was proven in vitro against Y-79 cells. Biocompatibility and toxicity studies were also performed in vivo on mice, where the PCB/IR PNPs were shown to be safe, making them a viable option for imaging-guided dual-cancer treatment [85].

### 5.2. PTT in Uveal Melanoma

In uveal melanoma PTT and theranostics, inorganic NPs have been used in conjunction to polymer-based nanoformulations, with nanogels in particular playing a key role. Nanogels or hydrogels consist of crosslinked polymeric networks and have the unique ability to undergo a phase transition, from a liquid or semi-liquid to solid, releasing their cargo as a response to specific stimuli. For instance, Li et al. created a Poly(N-isopropylacrylamide) (PNIPAM) hydrogel based on ultrasmall rare-earth NPs (RENPs), which have luminescent properties on their own, but were further decorated with ICG. They were then loaded with FA for target specificity as well as doxorubicin. The resulting nanogel (RENP-ICG@PNIPAM:Dox-FA) was dually responsive to both temperature changes and to glutathione (GSH), an antioxidant found in abundance in the tumor microenvironment due to its altered metabolism. Following the in vitro experiments, PTT with RENP-ICG@PNIPAM:Dox-FA was shown to have the best anti-tumor effect on OCM-1 cells compared to all other treatments, such as independent chemotherapy, PTT with ICG alone or the RENP-ICG@PNIPAM:Dox hydrogel without FA. Further experiments in vivo on a choroidal melanoma mouse model confirmed the synergy of combined treatment opposite plain doxorubicin and a non-targeted nanogel, with a significant reduction in tumor volume after 21 days of treatment. NIR-II imaging was also performed both in vitro and in vivo, confirming the excellent photothermal properties of the hydrogel while monitoring its distribution and biodegradability [11].

**Figure 5:**
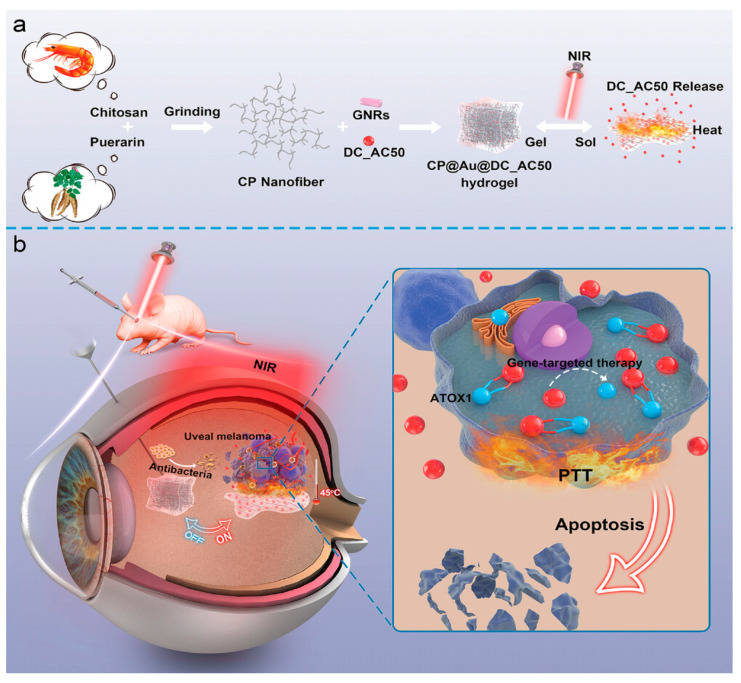
Schematic illustration of (**a**) the synthesis of the CP@Au@DC_AC50 hydrogel and its gel–sol transition via the NIR-light trigger and (**b**) its application in gene therapy/PTT with adjacent antibacterial properties for uveal melanoma. Reprinted/adapted with permission from [86] 19 February 2025, Wiley-VCH GmbH. Licensed under a Creative Commons Attribution 4.0 International License.

Shortly after the promising results of this research, another thermoresponsive hydrogel was presented by Wang et al. This self-assembling chitosan@puerarin(CP) gel incorporated AuNPs and DC_AC50, a gene-targeted drug that inhibits ATOX1, an antioxidant chaperone protein that is upregulated in uveal melanoma cells. Upon irradiation with an 808 nm laser, the light-responsive gold nanorods heated up, causing the nanogel to undergo a sol–gel transition, releasing the DC_AC50. Experiments with living mice confirmed the excellent photothermal and conversion properties of the hydrogel, as well as an “on–off” phenomenon through NIR-light triggers, allowing for further control of drug release and for multiple treatment sessions following a single intravitreal injection. The synergistic effect of PTT and the gene-targeted drug was also demonstrated against OCM-1 tumor-bearing mice. Combination therapy almost completely eradicated the tumor, as revealed by the clinical appearance, histological analysis and animal fluorescence imaging, where the signal was barely distinguishable. In contrast, the two treatments individually showed only a partial reduction, with the eyeballs still swollen and the fluorescent signal remaining strong. Lastly, the hydrogel was shown to be resistant to infections, which was attributed to the combination of PTT and the antibacterial properties of chitosan (Figure 5) [86].

**Figure 6:**
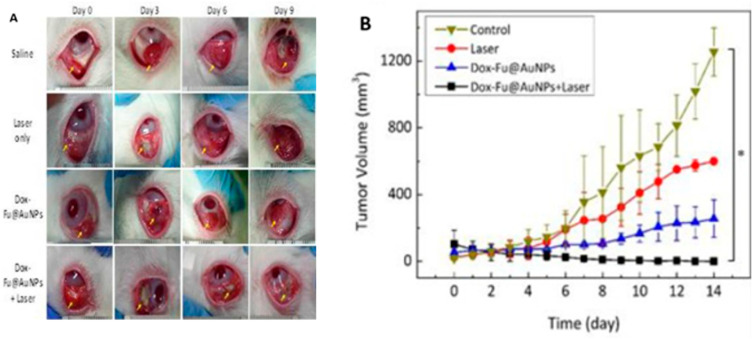
(**A**): Clinical presentation of the rabbit eye tumors for various groups. (**B**): Tumor volume over the course of 14 days for the same groups: control group (saline), laser (PTT without Dox-Fu@AuNP), Dox-Fu@AuNP (without laser) and Dox-Fu@AuNP+laser. Reprinted/adapted with permission from [87], 19 February, Impact Journals, LLC. Licensed under a Creative Commons Attribution 4.0 License. * *p* < 0.05.

AuNPs and polymers, specifically fucoidan, were also utilized by Kim et al. to conjugate doxorubicin for dual chemo-photothermal therapy. Fucoidan, a polysaccharide known for its anti-tumor and anti-inflammatory properties, was essential to ensure the successful conjunction of AuNPs and doxorubicin, since they were both positively charged. The final product of Dox-Fu@AuNPs was used both for PTT in vitro and in vivo, in tumor-bearing rabbits’ eyes, where the tumor was eliminated in its entirety within 6 days with no recurrence. In contrast, other treatments—Dox-Fu@AuNPs without a laser and PTT without NPs—only temporarily slowed tumor growth before it resumed increasing (Figure 6). As an additional benefit, photoacoustic imaging (PAI) was enhanced and a better visualization of the tumoral margins was achieved due to the light-absorbing properties of AuNPs. Therefore, this study not only harnessed the synergistic effects of chemotherapy and PTT, but also advanced in vivo image-guided treatment [87].

### 5.3. PDT in Retinoblastoma

Similar to PTT, NPs used in PDT aim to be multi-functional. For the treatment of retinoblastoma specifically, mesoporous silica NPs have been used in multiple experiments by the research group of the University of Montpellier. The group already demonstrated how MSNPs could be used for simultaneous PDT and chemotherapy in various types of cancer cells, by encapsulating a water soluble photosensitizer and camptothecin, a topoisomerase 1 inhibitor that works by destabilizing the S phase of the cell cycle and causing potentially lethal double-strand DNA breaks during DNA replication [88].

For retinoblastoma, they used the same method, adding mannose or galactose to target the receptors on Y-79 cells while also fluorescently labeling the MSNPs so that they could be monitored with confocal microscopy. Both groups performed significantly better at inducing cell death upon irradiation compared to MSNPs without camptothecin, or MSNPs with camptothecin but without the photosensitizer. The group then proceeded to experiments with biphotonic PDT by using a different photosensitizer. Biphotonic PDT, often referred to as two-photon PDT, utilizes the simultaneous absorption of two lower-energy photons rather than one higher-energy photon to activate the photosensitizers. This approach allows for deeper tissue penetration, as it uses NIR light instead of visible or ultraviolet light that is used in traditional PDT. It also offers increased precision and a three-dimensional spatial resolution, as only the small area where the two photons converge is activated. Moreover, because the photons are of lower energy, there are fewer scattering losses and reduced photodamage to surrounding tissues [89]. Indeed, biphotonic PDT proved to be more efficient, leading the group to adopt it for subsequent experiments where they focused on improving targeting specificity. They identified two subtypes of mannose receptors, MRC2 and CD209, which are overexpressed by Y-79 cells, and the MSNPs functionalized with those were the ones that achieved the highest cell death rates. Rhodamine was used to label the MSNPs so the cellular uptake through both endocytosis and co-location into the lysosomes could be tracked in real time with confocal microscopy, and the potential cytotoxic effect of PDT could also be monitored and adjusted. Overall, these experiments demonstrated promising outcomes, paving the way for simultaneous imaging, PDT, targeting and chemotherapy [90].

N’Diaye et al. experimented with hybrid NPs, composed of a polymer core and a phospholipid bilayer coating. This allowed them to encapsulate beta-lapachone (β-Lap) and the photosensitizer m-THPC, which are both lipophilic molecules, simultaneously in separate compartments. β-Lap is a naturally occurring quinone compound derived from the bark of the Lapacho tree that is activated by the enzyme NAD(P)H:quinone oxidoreductase (NQO1), which is often overexpressed in cancer cells. Once activated, it exerts its anticancer effects by inducing the generation of ROS. It was also hypothesized that the presence of ROS itself could further lead to the overexpression of NQO1, so that PDT-induced ROS could enhance the activation of β-Lap. Unfortunately, no such synergistic effect was made apparent by the experiments, but the combined therapies still showed an additive cytotoxicity on Y-79 cells, which allows for the use of lower, safer doses while maintaining the same level of effectiveness as single treatments [91].

### 5.4. Combined PTT/PDT

Although PTT and PDT have been at the forefront of the current anticancer research, both photothermal techniques have certain limitations when applied separately. On one hand, the overexpression of heat shock proteins in PTT can significantly suppress its cytotoxic effect, as their role is to protect other proteins from heat damage. On the other hand, PDT is overly dependent on oxygen, which can hinder its efficacy in hypoxic environments. By combining PTT and PDT, these limitations can be addressed, resulting in improved therapeutic outcomes. Given the pivotal role of NPs in both methods, it was a logical step to employ them in the integration of these two modalities.

**Figure 7:**
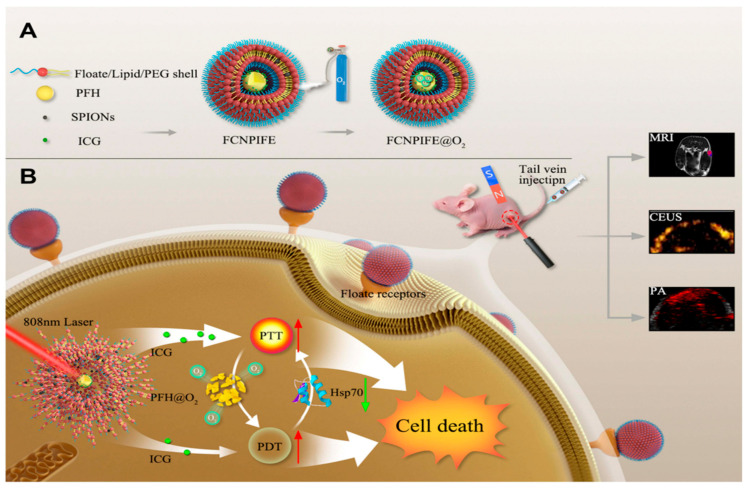
Schematic illustration of (**A**) the synthesis of FCNPIFEs and (**B**) US/PA/MR imaging-guided PTT/PDT for retinoblastoma. Reprinted/adapted with permission from [92], 19 February 2025, Dove Medical Press Limited,. Licensed under a Creative Commons Attribution 3.0 International License.

With this in mind, Zheng et al. formulated superparamagnetic cationic nanoliposomes loaded with ICG and perfluorohexane (PFH), creating a versatile platform for the treatment of retinoblastoma. PFH is a perfluorocarbon that has been widely used in nanomedicine due to its phase changing ability from liquid to gas upon the absorption of enough energy, and its oxygen-carrying and releasing function, which can reinforce PDT in hypoxic regions. Superparamagnetic Iron Oxide NPs (SPIONSs) were embedded within the lipid bilayer, along with folate for targeting, while the ICG and PFH were encapsulated in the core. SPIONSs served not only as contrast agents for MRI, but also for targeting through the application of an external magnet. Similarly, ICG was utilized for PAI as well as a photosensitizer for PTT and PDT. This approach requires a single excitation source, an 808 nm laser, which largely simplifies the process of combining the two photothermal therapies. Although admittedly ICG-based PDT was challenging in the hypoxic tumor environment, PFH’s oxygen supply compensated for it. PFH’s phase change upon irradiation and the resulting microbubbles facilitated US imaging. The dual-targeted (folate/magnetic) nanoliposomes were tested in vitro as well as in Y-79 tumor-bearing mice and demonstrated the synergy of combined PTT/PDT along with successful multimodal US/PA/MR imaging (Figure 7) [92].

**Figure 8:**
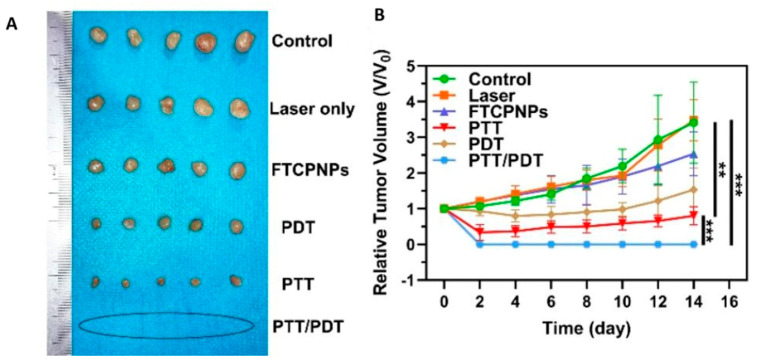
(**A**) Photographs of the dissected tumor for each group. (**B**) Line graph of relative tumor volume in relation to time for each group. Reprinted/adapted with permission from [93], 19 February, Springer Nature. Licensed under a Creative Commons Attribution 4.0 International License. ** *p* < 0.01, *** *p* < 0.001).

A similar nanoplatform was introduced by Huang et al. for uveal melanoma theranostics. They chose PLGA NPs, which they loaded with chlorin e6 (Ce6) and coated with FeIII–tannic acid (FeIII-TA). Ce6 is a chlorophyll derivative that serves as a PDT photosensitizer, but as is often the case with photosensitizers, its use is limited due to its tendency to aggregate in aqueous solutions. FeIII-TA is a complex formed between iron in its ferric state (FeIII) and tannic acid, a natural polyphenol, which exhibits excellent photothermal and paramagnetic properties, enabling its use in PAI and PTT as well as MRI. Since Ce6 and FeIII-TA are optimally excited at different wavelengths, the use of both a 660 nm laser for PDT and an 808 nm laser for PTT was imperative. Following the in vitro experiments on C918 cells that confirmed the synergistic effect of dual photothermal therapy, the resulting FTCPNPs (FeIII-TA/PLGA/Ce6 NPs) were tested in a mouse model. The accumulation of NPs in the tumor region was confirmed through dual PA/MR imaging, which helped define the therapeutic window by pinpointing the moment of peak accumulation. Photothermal imaging was employed as well to monitor the temperature during PTT and ensure no damage was inflicted to the surrounding tissues. The results of this study are presented in Figure 8, where tumor-bearing mice were randomly divided into 6 groups: the control group that received no treatment; the laser only group that received irradiation with a 660 nm laser; the FTCPNP group, where FTCPNPs were injected but not irradiated; the PTT group where FTCPNPs were used for PTT with an  808 nm laser; the PDT group where FTCPNPs were used for PDT with a  660 nm laser and the PTT/PDT group, where FTCPNPs were irradiated by both 808 nm and 660 nm lasers for combined PTT/PDT. Remarkably, the PTT/PDT group achieved complete tumor eradication in 14 days [93]. This study serves as an exemplification of how PTT and PDT can be complementary to each other and how NPs make their pairing possible.

## 6. Nanoparticles in Brachytherapy

Brachytherapy, also known as episcleral plaque radiotherapy, is a specialized form of radiation therapy used to treat intraocular tumors that involves suturing a radioactive plaque on the sclera. This permits the delivery of highly concentrated radiation to the tumor site, the dose of which depends on the type and amount of the radioactive seeds and can be further adjusted by the length of time. It is the most commonly used eye sparing treatment for uveal melanoma, with a survival rate similar to enucleation. It can also be used for retinoblastoma as long as there is no vitreous seeding [3].

Several isotopes have been used in brachytherapy, such as cobalt-60, iodine-125 (^125^I), iridium-192, palladium-103 (^103^Pd) and ruthenium-106 (^106^Ru), but clinical practice has mainly settled on ^125^I and ^106^Ru due to their good dosage distribution and availability. Additionally, the emission of gamma rays from ^125^I offers an advantage in treating larger tumors, as it provides greater tissue penetration compared to the beta-emitting ^106^Ru [94].

Nanotechnology can improve brachytherapy by enhancing the radiation dose and improving targeting to minimize the damage inflicted on surrounding healthy tissues. AuNPs act as potent radiosensitizers by leveraging their high atomic number (Z = 79) that allows them to efficiently absorb and scatter the radiation, and their strong photoelectric effect that produces secondary photo/Auger electrons to cause further damage to the cancer cells. They are also stable, can easily be functionalized with drugs and targeting ligands and their small size permits intratumoral accumulation.

AuNPs have been previously proven to sensitize cutaneous melanoma B16F10 cells to radiation in vivo [95] and to disrupt tumor vasculature when combined with brachytherapy [96]. Building on these principles, AuNPs have been investigated as a treatment for uveal melanoma. Asadi et al. utilized them in a Monte Carlo model of the human eye, demonstrating that the tumor site absorbed significantly more radiation in the presence of AuNPs than with ^125^I irradiation alone. This finding suggests that the required therapeutic dose can be achieved with shorter brachytherapy sessions, reducing the potential damage to surrounding healthy tissues [97].

Further evidence of dose enhancement with gold nanospheres came from another Monte Carlo model using ^103^Pd as the radioisotope, where higher concentrations of AuNPs directly correlated to higher doses intratumorally and the shorter time required to administer the desirable dose [98]. However, a comparative study evaluating both ^103^Pd and ^125^I in NP-assisted brachytherapy revealed a more pronounced dose enhancement factor with ^125^I, solidifying its position as the preferred isotope [99].

Monte Carlo models were also used to study the parameters that affect dosimetry, such as the size and concentrations of AuNPs and low- or high-energy photon sources. It was concluded that higher concentrations and AuNPs with larger diameters were optimal for dose enhancement, while the efficacy of low-energy sources was highlighted [100].

The AuNP-assisted brachytherapy approach was subsequently escalated to in vitro experiments with choroidal melanoma cells. After being exposed to various concentrations of AuNPs, as well as radiation alone, cell viability was assessed throughout a 7-day period. While lower concentrations had no significant cytotoxic effect over the first three days compared to radiation without AuNPs, cell death reached 30% by day 7 as opposed to merely 10% with plain radiation. Higher concentrations achieved even more impressive results, with over 70% of cell apoptosis by the seventh day [101].

The concern of whether NPs are indeed sufficiently selective was also addressed by Kanavi et al. who investigated the distribution of AuNPs ex vivo in an enucleated human eye with choroidal melanoma. They found that the AuNPs did not reach extratumoral areas, but instead dispersed within the tumor and highly infiltrated the intratumoral vascular endothelial lining, which is on par with their ability to disrupt the malignant vasculature [102]. The dose distribution itself in brachytherapy with ^125^I seeds was also measured in a model eyeball, and the presence of AuNPs resulted in a higher dose within the tumor and lower dose in the healthy tissues compared to plain brachytherapy [103].

However, in a later study with a ^106^Ru plaque in a Monte Carlo model, optimal dose enhancement was achieved for short distances from the plaque surface, while for more than 2mm, the dose enhancement factor dropped, and more radiation was accumulated in the sclera. The group hypothesized that this phenomenon may be due to high-energy electrons interacting with the heavy gold nuclei and releasing Bremsstrahlung photons that stray from the intended target and can reach healthy tissues. They concluded that while AuNPs can ameliorate dose distribution for superficial tumors, they may not be a wise choice for deeper-reaching tumors [104].

Other ways in which NPs can contribute to brachytherapy apart from dose enhancers are by acting as radiation shields. In a recent study, magnetite NPs suspended in a silicon polymer were used to formulate high-density ferrofluids. A modified magnetic plaque was then used to attract ferrofluids to the tumor surface, directing the radiation to the target while minimizing the exposure of the neighboring tissues [105]. NP-assisted brachytherapy also provides the opportunity for combination therapy. For example, ^125^I brachytherapy with AuNps and simultaneous ultrasonic hyperthermia was tested on a rabbit model by Moradi et al. for the treatment of retinoblastoma. All different combinations of brachytherapy, phototherapy and AuNPs, as well as the respective monotherapies were tested, and the group that involved all three performed the best, thus proving their synergistic effect [106].

Alternatively to brachytherapy, external beam radiotherapy has been used in conjunction with NPs, which delivers a lower total dose and has lower dose enhancement, but has the advantage of being noninvasive [107]. Finally, Altundal et al. experimented not only with AuNPs for dose enhancement, but also with carboplatin NPs for adjuvant chemotherapy in both choroidal melanoma and retinoblastoma [108].

## 7. Nanoparticles in Gene Therapy

NP-mediated gene delivery has been explored as an alternative to viral carriers for the treatment of ocular diseases. While viral vectors, such as adeno-associated viruses (AAVs), have demonstrated high efficiency in gene transduction and integration, they present several significant disadvantages. One major concern is the triggering of immune responses, which can lead to inflammation and the production of neutralizing antibodies, particularly with repeated treatments. These immune reactions not only reduce the efficacy of the therapy, but may also result in serious side effects. Additionally, viral vectors are constrained by their limited cargo capacity, with recombinant AAV vectors reportedly being able to accommodate inserts up to 4.7 kb, making them unsuitable for larger genes [109,110]. Other limitations include cell tropism, which restricts the types of cells that can be targeted, and the random integration of viral DNA into the host genome, raising concerns about potential oncogenesis. In some clinical trials, the use of viral vectors has indeed led to severe outcomes, such as cancer or mortality due to a systemic inflammatory response [111,112]. Despite their promise, these drawbacks necessitate careful consideration when using viral vectors in gene therapy and highlight the need for safer alternatives [113,114,115].

In search of non-viral methods for gene therapy, different types of NPs have been studied, with the most prominent being liposomes, polymeric NPs and combinations of them. The qualities that are sought after for an ideal vector are a high transduction efficiency, capacity to carry large sequences of nucleic acids, targeted transgenesis and a safe profile at a reasonable cost. The main physiological challenge when selecting a vector is the penetration of the cell membrane. As discussed, the key factors for permeating the cell membrane are size (ideally less than 200 nm), surface charge and surface chemistry. Positively charged NPs can enter the negatively charged cell membrane through endocytosis, mainly macropinocytosis or clathrin-mediated endocytosis. Functionalizing their surface with ligands that bind to membrane receptors also promotes endocytosis. For directly acting nucleic acids, such as messenger RNA (mRNA) or small interfering RNA (siRNA), it suffices to enter the intracellular space while DNA sequences have the additional challenge of bypassing the nuclear membrane [110,116].

### 7.1. Lipid NPs in Gene Therapy

Lipid NPs have several advantages and can be modified to accommodate the needs of an effective gene carrier. They can be composed from cationic or ionizable lipids, PEGylated lipids and structural lipids that self-assemble. Apart from facilitating the entry inside the cell, ionizable lipids are responsible for binding nucleic acid and protecting it from endosomal lysis, while PEG ensures the stability of the formulation [115]. Such LNPs were used for the delivery of mRNA to the posterior segment of the eye in vivo by Patel et al. to treat retinal degeneration [117].

Further gene transfer has also been achieved with DNA plasmids in Rs1h-deficient mice suffering from juvenile retinoschisis, a genetic degenerative disorder of the retina. Apart from the RS1 gene, the EGFP plasmid was also loaded in the LNPs to induce the expression of the Enhanced Green Fluorescent Protein (EGFP) for visualization and tracking purposes [118,119,120]. The successful gene transduction in retinal cells along with studies that employ gene therapy for cancer treatment [121], and even the simultaneous encapsulation of chemotherapeutic agents [122], are very promising for the use of LNPs as gene carriers in the treatment of retinoblastoma. In fact, in vivo testing has already started, as Tabatabaei et al. (2019) formulated cationic LNPs to co-deliver melphalan and miR-181a, a microRNA that downregulates the anti-proliferative gene MAPK1 and the anti-apoptotic gene Bcl-2, while boosting the expression of pro-apoptotic gene BAX in retinoblastoma cells [123]. Another multimodal approach was executed by Wu et al. for simultaneous imaging and laser-activated gene release. They introduced cationic NPs composed of a lipid shell modified with FA for targeting, inside which they encapsulated liquid PFP and ICG for PA and US imaging. The cationic surface of the NPs facilitated the attachment of the HSV-TK/GCV suicide gene system, a set of genes that encode the herpes simplex virus type thymidine kinase (HSV-TK) and its prodrug ganciclovir (GCV), the combination of which is highly cytotoxic. The recombinant pDNA of GFR was also added for tracking. Laser irradiation was then employed to increase cell membrane permeability, enhancing DNA uptake. Thus, upon NIR laser exposure, Rb cells demonstrated efficient transfection, as evidenced by the strong GFP signal and a significant increase in apoptotic cell death [124].

Of course, LNPs do not come without some disadvantages. Due to their charge, when used repetitively they can induce significant immune and inflammatory responses compared to neutral NPs or interact with negatively charged extracellular components and form aggregates. To avoid this, PEG or polysaccharides, such as dextran or hyaluronic acid, can be added, and they have been shown to facilitate their course through the vitreous body to better reach the retinal layers [119,120,125].

Moreover, the condensation of nucleic acid is desirable in order to achieve a bigger load, but it can lead to problematic transfection if performed at an excessive degree, as it inhibits its release from the complex [126]. To address the low transfection capacity, the use of peptides, such as the TAT2 peptide or protamine, has been implemented in in vivo experiments. Rudolph et al. showed that the compaction of pDNA with TAT2 before loading it to the SNL vectors improved the gene transfer efficiency to bronchial epithelial cells. Delgado et al., who experimented with ARPE-19 cells, specifically for the treatment of X-linked juvenile retinoschisis, opted for protamine to stabilize pDNA. This method, along with the addition of a dextran coating, significantly enhanced the expression of retinoschisin and EGFP in vitro and subsequently in vivo [120,127].

### 7.2. Polymeric NPs in Gene Therapy

Another promising non-viral vector is compacted DNA/RNA–polymeric NPs. These nanoformulations that typically range from 1 to 100 nm are usually composed of a nucleic acid segment compacted with a polycationic polymer [113]. Polymers have been studied since the beginning of the millennia and different types of polymers, such as carbohydrate polymers and dendrimers, have been used as gene delivery carriers in cancer therapy [128].

For ophthalmic cancers, the main focus is gene delivery to the retina, which has also been widely studied. For example, plasmid DNA (pDNA) for the Enhanced Green Fluorescent Protein (EGFP) compacted with PEG-substituted lysine peptides was successfully transferred to the retina by Farjo et al. in 2006 [114]. Other early studies explored co-polymers, such as PLA and PLGA, for the same purpose and observed the greatest uptake by the RPE [129,130]. PLGA is negatively charged in a neutral pH and only becomes positively charged in the acid environment of the endolysosomal intracellular sites. Therefore, it typically needs to be enhanced with other cationic molecules, such as chitosan or PEI, which have excellent compatibility [131]. Such chitosan–PLGA NPs have already been applied to the treatment of retinoblastoma by delivering etoposide to Y-79 cells, as mentioned earlier [17], while they have also successfully delivered pDNA to the retina for the inhibition of neovascularization [132,133]. Taking this into consideration, PLGA-assisted gene delivery in retinoblastoma can be envisioned in the future.

The other categories of polymers, such as polysaccharides, e.g., chitosan, dextran and hyaluronan, as well as dendrimers, can also be used in gene delivery independently of PLGA. Polysaccharides are naturally occurring molecules, so they stand out for their biocompatibility, and they have various functional groups that makes them easily modifiable [134]. They have already been employed for chemotherapy delivery in retinoblastoma, so it is auspicious that their use can be extended in gene delivery [37]. For example, the compacted pDNA of Green Fluorescent Protein (GFP) was loaded in glycol chitosan as early as 2014 by Mitra et al. and was shown to reach the RPE in vivo [135].

As far as dendrimers are concerned, PEI holds a pivotal position in gene delivery, boasting notable advantages, such as a cationic charge that facilitates binding and condensing the negatively charged amino acids, and the ability to escape the endosome due to the proton sponge effect. The amino groups of PEI act as buffers to the acidic environment of the endosome by absorbing protons, leading to the osmotic swelling of the endosome and eventually its rupture, so that the cargo can escape to the cytoplasm [136].

In ophthalmology, there are instances of formulations consisting of PEI that have been used for DNA delivery to retinal ganglion cells [137], as well as other dendrimers, such as PLL-LAA, for reaching the RPE [138]. The PEGylation of PEI has been previously discussed as a method of neutralizing its charge, thus rendering it less cytotoxic. Wang et al. applied this technique and created the hyperbranched-star PEI, grafted with PEG (PEG-g-PEI), which can effectively condense and carry genes. Specifically, pDNA encoding-enhanced red fluorescent protein (RFP) and GFP were used to study the cellular uptake of the vectors by retinoblastoma cells and gene transfection efficiency in vitro. Compared to other non-PEGylated PEI-based vectors, which failed to induce RFP expression, the hyperbranched-star PEG-g-PEI was successful in RFP transfection, accomplished higher GFP expression and had a markedly improved biocompatibility and safety profile [139].

In another study, pDNA was compacted in dendrimer NPs to transfect the human choroidal melanoma OCM-1 cell line. The recombinant pDNA that was used co-expressed the tumor necrosis factor-α (TNF-α) and the herpes simplex virus thymidine kinase (HSV-TK), which leads to cancer cell apoptosis while sparing healthy cells through a process called suicide gene therapy, first described by Moolten [140]. After the transfection, OCM-1 was also irradiated with 2 Gy ^125^I, which led to increased gene expression [141].

Last but not least, combinations of the different types of NPs, such as lipid and polymeric NPs, are possible. Tan et al. experimented with PAMAM dendrimers, which functionalized with three different amino acids to improve biocompatibility and cellular uptake. DNA and a nuclear localization signal (NLS), which is a short sequence of amino acids that facilitates the delivery of DNA to the cell nucleus, were integrated to the dendrimers, and the entire complex was encapsulated in a lipid bilayer. The lipids chosen were pH-sensitive, so that the lipid bilayer could fuse with the endosomal membrane upon exposure to the acidic environment of the endosome, releasing the cargo to the cytoplasm. Finally, hyaluronic acid-1,2-dioleoylphosphatidylethanolamine (HA-DOPE) was used for the outer coating, as HA targets the CD44 receptor of the retinal cells. In in vitro studies with ARPE-19 cells, the nanocarriers demonstrated a good endosomal escape ability, penetration of the nucleus and transfection efficiency. Similar results were accomplished in vivo with minimal cytotoxicity [142].

### 7.3. Inorganic NPs in Gene Therapy

Gene therapy research is not limited to organic NPs alone. Inorganic NPs are also gaining popularity, with AuNPs having been extensively studied as carriers for nucleic acids [143,144,145,146]. AuNPs have been established as highly versatile NPs, one of their key advantages being the ease of modifying their surface. In fact, it has been shown that the surface modification of AuNPs with cationic NPs, such as PEI, creates a mutually beneficial relationship: the PEI enhances the binding ability of nucleic acid to the AuNPs, while the AuNPs mitigate PEI’s inherent cytotoxicity. Such complexes have shown promise in anti-oncogenic gene therapy [147].

In regard to those principles, Mitra et al. used PEI-capped AuNPs to deliver siRNA to retinoblastoma cells. The AuNP-PEI nanocarriers were conjugated with an EpCAM antibody to target EpCAM-expressing cells. EpCAM not only serves as a target to detect malignant cells, it also plays a crucial role in tumor cell proliferation, metastasis and survival [148]. Thus, the group used EpCAM siRNA to silence the EpCAM gene and disrupt its signaling pathway in vitro in Y-79 cells, while proving that siRNA loaded in EpCAM-AuNP-PEI nanoconjugates was effective in half the amount of naked siRNA required for the same result. They also demonstrated that EpCAM-AuNP-PEI was safe for healthy cells [149].

Posch et al. focused on the GNAQ mutations that are an oncogenic and metastatic driver in a high number of uveal melanoma cases. They designed AuNPs to both detect mutated GNAQ mRNA and to silence them using siRNA. For detection, the AuNPs were functionalized with oligonucleotides that would structurally change upon binding to the mutated mRNA, resulting in a detectable fluorescent signal in confocal microscopy. For siRNA delivery, they developed a novel release system through the modification of siRNA-AuNPs with a dithiolane moiety and disulfide groups, which could release the siRNAs faster and more effectively in the presence of the antioxidant GSH. The efficacy and selectivity of the siRNA-AuNPs was proved in the uveal melanoma cell line OMM1.3, whose viability steeply declined due to the disruption of GNAQ signaling, whereas a minimal impact was observed in healthy cells or cells without the specific mutation [150].

It is also worth mentioning that inorganic NPs have been used for molecular therapy that falls outside the strict definition of gene therapy as carriers of peptides and even multimodal applications. Kalmodia et al. designed AuNPs conjugated with the anti-HDM2 peptide, a peptide that inhibits the interaction between the tumor suppressor protein p53 and its negative regulator, human double minute 2 (HDM2), which degrades p53 and limits its tumor-suppressing activities. In vitro experiments on Y-79 cells demonstrated that AuNP-HDM2 could restore the functions of p53, and identified p53-mediated tumor suppressor miRNAs as another potential target for the future research [151].

Wang et al. also used AuNPs, which they conjugated with iron oxide, to create magnetic, hollow, mesoporous gold nanocages (AuNCs–Fe_3_O_4_) to deliver the muramyl dipeptide (MDP) to Rb cells. Apart from the MDP, which is an immunomodulator used in cancer therapy, they added PFP to enhance US imaging, as well as low-intensity focused ultrasound (LIFU) therapy. The resulting AuNC–Fe_3_O_4_/MDP/PFP NPs could be used for combined LIFU/immunotherapy while being guided simultaneously by multimodal PA/US/MR imaging [152].

Finally, other types of inorganic NPs, such as MSNPs and iron oxide NPs, have been used for the delivery of nucleic acids, on their own [153,154,155,156] or in combination with organic NPs [157,158,159,160]. With their applications extending in oncology [158,161], there is the possibility that they will be adapted for ophthalmic uses and ocular cancers in particular, in the near future.

## 8. Safety and Regulatory Concerns

As with every emerging and rapidly growing field in medicine, safety and toxicity issues are of primary concern. As materials shrink to nano-dimensions, they acquire a new set of physicochemical properties, very different from their bulk counterparts, which are not yet completely understood. Therefore, although the material itself is an important factor of toxicity, there are also other determining factors, most notably size, shape and charge. Smaller-sized NPs are generally considered more toxic due to their higher surface-area-to-volume ratio that allows them to interact more readily with biological systems and their increased ability to bypass biological barriers, including the cellular membrane. They also pose a greater risk of genotoxicity, which refers to their potential to cause damage to genetic material [162]. As far as surface charge is concerned, charged NPs are more likely to elicit an immune response, induce ROS production or interfere with the mitochondria [163]. Positively charged NPs in particular are more attracted to the negatively charged cellular membranes, which increases cellular uptake, while some can even escape the lysosomes and enter the nucleus, making them more likely to induce DNA damage [164].

Nonetheless, the mere endeavor to study nanotoxicity can be challenging. The ADME (absorption, distribution, metabolism, excretion) profile of nanomedicines, which is the main tool for evaluating safety, remains unclear due to the difficulty in detecting nanosized molecules to monitor biodistribution, accumulation and excretion pathways [165]. For example, the increased mobility and permeation of biological barriers can be detrimental in the case of crossing the blood–brain barrier, while renal and hepatic accumulation are equally alarming. ADME profiles can also vary significantly with slight changes in NP parameters, requiring extensive studies for different combinations. NP characterization is further complicated by changes in morphology during storage or in vivo administration, the lack of standardized methods and unpredictable interactions, like protein corona formation, which alters their behavior in vivo [166,167]. Poor batch-to-batch reproducibility, aggregation and instability add to manufacturing and regulatory challenges, especially for multifunctional and hybrid NPs [165,168,169,170].

All the above factors contribute to the difficulty of setting clear regulatory mechanisms in place and delay the translation of nanomedicine from preclinical research to clinical use. In addition, NPs are not well-defined and there is currently no global consensus on their classification. The issue is magnified with multifunctional NPs, which may combine diagnostic, therapeutic and targeting capabilities within a single platform, blurring the boundaries between drugs, devices and biologics, and requiring evaluation under multiple regulatory frameworks.

Despite the challenges, several nanoformulations have become available in the market after extensive testing that ensures their safety. In ophthalmology, formulations for all sorts of applications, from dry eye disease (ex. Cequa^®^; Sun Pharmaceuticals Inc., Mumbai, India, Restasis^®^; AbbVie Inc., North Chicago, IL, USA, Ikervis^®^; Santen Pharmaceutical Co., Ltd., Osaka, Japan, Lacrisek^®^ BIOOS Italia, Pisa, Italy), glaucoma (Xelpros^®^; Sun Pharmaceutical Inc., Mumbai, India) and infections (AzaSite^®^; Thea Pharma Inc., Lexington, MA, USA) to posterior segment diseases, such as uveitis (Trivaris^®^; Allergan, Inc., Irvine, CA, USA, Retisert; Bausch & Lomb Inc., Rochester, NY, USA), macular oedema (Ozurdex^®^; Allergan, Inc., Irvine, CA, USA, Iluvien^®^;Alimera Sciences, Inc., Alpharetta, Georgia) and wet AMD (Macugen^®^; Eyetech Pharmaceuticals Inc. and Pfizer Inc., New York, NY, USA, Visudyne^®^; Cheplapharm Arzneimittel GmbH, Greifswald, Germany), have been FDA or EMA approved and are available on the market [12]. Similarly, nano-delivery systems of chemotherapeutic drugs, such as Vincristine and doxorubicin, have been approved for other types of cancers, paving the way for their potential use in ophthalmic oncology and facilitating their progression to clinical trials. Last but not least, AU-011, designed specifically for retinoblastoma, has made it into phase 2 trials. Many more preclinical studies are underway, with a comprehensive table of those mentioned in this review provided in Supplementary Materials Table S1.

## 9. Conclusions

Nanomedicine has made significant contributions to ophthalmology and is now expanding into the field of ocular oncology, transforming both diagnosis and treatment. Over the past two decades, advancements in NP-based drug delivery, imaging and theranostics have provided promising alternatives to conventional therapies, overcoming key challenges, such as ocular barriers and systemic toxicity. With several preclinical studies, as well as some transitioning into clinical studies, nanomedicine paves a new path for the management of ophthalmic cancers, with multimodal applications and targeted and personalized treatment being at the forefront. Nevertheless, further research and cautious, comprehensive regulations and frameworks are essential to ensure patient safety and optimal quality of care. With continued innovation and rigorous scientific validation, nanomedicine holds the promise of transforming the future of ocular oncology.

## Data Availability

No new data were created or analyzed in this study. Data sharing is not applicable to this article.

## Supplementary Materials

The following supporting information can be downloaded at: https://www.mdpi.com/article/10.3390/cancers17071186/s1; Table S1: Comprehensive table of studies on nanoparticle applications in ocular cancer management.

## Abbreviations

The following abbreviations are used in this manuscript:

EPR	Enhanced Permeability and Retention
PLGA	Poly(lactic-co-glycolic acid)
PAMAM	Poly(amidoamine)
NP	Nanoparticle
SLN	Solid Lipid Nanoparticle
MSNP	Mesoporous Silica Nanoparticle
FA	Folic Acid
CE-MIP	Carboplatin and Etoposide Molecularly Imprinted Polymer
VCR	Vincristine
ROS	Reactive Oxygen Species
PEG	Polyethylene Glycol
NIR	Near-Infrared
ICG	Indocyanine Green
PFP	Pentafluropentane
MDR	Multidrug Resistance
MAPK	Mitogen-Activated Protein Kinase
p53	Tumor Protein p53
SLT	Selective Laser Trabeculoplasty
OCT	Optical Coherence Tomography
PAI	Photoacoustic Imaging
SPIONS	Superparamagnetic Iron Oxide Nanoparticle
TA	Tannic Acid
Ce6	Chlorin e6
PDT	Photodynamic Therapy
PTT	Photothermal Therapy
GFP	Green Fluorescent Protein
HSV-TK	Herpes Simplex Virus Thymidine Kinase
GCV	Ganciclovir
BAX	Bcl-2 Associated X Protein
MAPK1	Mitogen-Activated Protein Kinase 1
NLS	Nuclear Localization Signal
TNF-α	Tumor Necrosis Factor-Alpha
TK	Thymidine Kinase
^125^I	Iodine-125
^106^Ru	Ruthenium-106
^103^Pd	Palladium-103
AuNP	Gold Nanoparticle
LIFU	Low-Intensity Focused Ultrasound
MDP	Muramyl Dipeptide
