# Supplementary material for: The Contribution of Nanomedicine in Ocular Oncology
Source: Cancers (Basel). 2025 Mar 31;17(7):1186. doi: 10.3390/cancers17071186 (PMC11987995; doi:10.3390/cancers17071186)
Supplement: Supplementary file 1 [file cancers-17-01186-s001.zip › cancers-3529340-supplementary.pdf]

Supplementary Material

Table S1. Comprehensive Table of studies on nanoparticle applications in ocular cancer management

Type of NP	Short Description	Function	Type of Trial	Type of Cancer	Reference
**PLGA**	upregulation of apoptotic gene activity in Y-79 cancer cells with etoposide-PLGA NPs	chemo with etoposide	in vitro	RB	[16]
**PLGA**	etoposide-PLGA NPs for chemotherapy	chemo with etoposide	in vitro	RB	[17]
**SLN**	etoposide-loaded SLNs improve bioavailability of etoposide via intravitreal injection	chemo with etoposide	in vivo	RB	[18]
**PAMAM dendrimers**	subconjunctival administration of carboplatin-loaded PAMAM for murine RB	chemo with carboplatin	in vivo	RB	[19]
**MSNPs**	carboplatin EpCAM-MSNPs for RB treatment	chemo with carboplatin	in vitro	RB	[20]
**proteinic NPs (apotransferrin and lactoferrin)**	proteinic NPs as carriers for carboplatin, cytotoxicity in Y-79 cells - pH dependant release	chemo with carboplatin	in vitro	RB	[21]
**lactoferrin protein Nanoparticles**	proteinic NPs as carriers for etoposide and carboplatin for Y-79 cells	chemo with etoposide and carboplatin	in vitro	RB	[22]
**PLGA**	PLGA nanoparticles with carboplatin and etoposide for RB - subconjunctival inj.	chemo with etoposide and carboplatin	in vivo	RB	[23]
**liposomes**	safety and toxicity profile of liposomal vincristine sulfate in patients with metastatic uveal melanoma	chemo with vincristine	Clinical trial	UM	[24]
**liposomes**	liposomal vincristine in uveal melanoma	chemo with vincristine	Phase 2 trial	UM	[25]
**liposomes**	liposomal vincristine in RB	chemo with vincristine	Phase 3 trial	RB	[26]

**liposomes**	liposomal vincristine in RB	chemo with vincristine	Phase 3 trial	RB	[27]
**polymeric/SPIONs**	Vincristine-loaded Pluronic f127 polymer-coated magnetic nanoparticles conjugated with FA and transferrin for RB chemo and hyperthermia	chemo with vincristine/hyperther mia	in vitro	RB	[28]
**micelles**	PLGA-PEG-FOL micelles in a thermoresponsive gel for sustained doxorubicin delivery to retinoblastoma cells	chemo with doxorubicin	in vitro	RB	[30]
**chitosan NPs**	folate-conjugated doxorubicin-loaded chitosan nanoparticles for targeting RB cells	chemo with doxorubicin	in vitro	RB	[31]
**CeO2 nanoceria**	Doxorubicin and AMD11070 loaded nanoceria for RB	dual chemo	in vivo	RB	[32]
**PLGA NPs**	melphalan loaded PLGA nanoparticles with peptide-modified surface for retinoblastoma	chemo with melphalan	in vitro	RB	[33]
**chitosan NPs**	chitosan-alginate melphalan NPs for topical administration in RB	chemo with melphalan	in vivo	RB	[34]
**chitosan NPs**	two studies, topotecan-loaded chitosan NPs in retinoblastoma	chemo with topotecan	in vivo	RB	[36]
**MSNPs**	topotecan-loaded MSNPs, which they decorated with folic acid	chemo with topotecan	in vivo	RB	[38]
**micelles**	dasatinib-loaded polymeric micelles for intravitreal inj in proliferative vitreoretinopathy in mice	chemo with Dasatinib	in vivo	UM	[42]
**PLGA NPs**	Folate/nutlin-3a/Curcumin NPs for RB	chemo with curcumin	in vitro	RB	[46]
**CO-HA hydrogel**	In situ gel curcumin loaded NP for UM	chemo with curcumin	in vitro	UM	[47]
**AuNPs**	albumin-stabilised gold nanoclusters loaded with AZD8055, an mTOR kinase inhibitor	chemo with TOR kinase inhibitor	in vivo	UM	[48]
**PLGA**	PLGA NPs loaded with Oleanolic (OA) or its isomer, ursolic acid (UA)	chemo with triterpenes	in vitro	RB	[51]
**PAMAM dendrimers**	Methotrexate-loaded PAMAM functionalized with FA	chemo with Methotrexate	in vitro	intraocular lymphoma	[52]
**PAMAM dendrimers**	Methotrexate-loaded PAMAM functionalized with FA	chemo with Methotrexate	in vivo	intraocular lymphoma	[53]

**AuNPs**	nanorods with antibodies specific for GLUT-1 for OCT imaging	OCT imaging	in vitro	Conjunctival squamous cell carcinoma	[56]
**AuNPs**	gold nanocages for PAI imaging of UM in porcine eyes	PAI imaging	ex vivo	UM	[57]
**MSNPs**	mannose-functionalized fluorescently labelled MSNPs to target Rb cells	fluorescent confocal microscopy	in vitro	RB	[58]
**Iron Oxide NPs**	dextran-coated iron oxide nanoparticles (DCIONs) to induce magnetic hyperthermia	hyperthermia	in vitro	RB	[64]
**AuNPs**	Ultrasonic hyperthermia with AuNPs for RB cells	hyperthermia	in vitro	RB	[65]
**AuNPs**	PEG-ylated gold nanorods coated with EpCAM antibodies and femtosecond lasers	hyperthermia	in vitro	RB	[67]
**AuNP and AuAgNPs**	hyperthermia with nanosecond laser and AuNP and AuAgNPs for Y79 cultures in a vitreous phantom model	hyperthermia	in vitro/phantom model	RB	[68]
**liposomes**	mannosylated porphyrins in liposomes for PDT	PDT	Rb cel membrane model	RB	[69]
**TiO2**	TiO2 and Ce- doped TiO2 for PDT in Rb	PDT	in vitro	RB	[70]
**vectosomes**	vectosomes activated upon illumination inhibit OCM-1 cell proliferation	Light-activated delivery	in vivo	UM	[71]
**VPL (virus-like particle)**	AU-011, light-activated NP for UM	Light-activated immunotherapy	Phase 2 trial	UM	[76]
**liposomes**	PDT with Visudyne, various clinical studies	PDT	Clinical trials	UM/RB	[77–81]
**liposomes**	ICG-liposomes for image-guided PTT	imaging/PTT	in vivo	RB	[82]
**liposomes**	ICG-liposomes with DOX, decorated with FA	imaging/PTT/chemo	in vivo	RB	[83]
**QD/lipid NP**	melphalan and BPQD in lipid nanoparticles	PTT/chemo	in vivo	RB	[84]
**PLGA/PCL NPs**	PLGA and PCL NPs, conjugated with NIR dye and Palbociclib	PTT/chemo	in vivo	RB	[85]

**PNIPAM hydrogel/RENP**	dually responsive nanogel to temperature changes and glutathione, with DOX, for PTT	imaging/PTT/chemo	in vivo	UM	[11]
**chitosan@puerarin hydrogel/AuNPs**	thermoresponsive nanogel incorporating AuNPs and DC_AC50	PTT/chemo/antibacterial	in vivo	UM	[86]
**AuNPs/polymer**	AuNPs/fucoidan conjugatd with DOX	imaging/PTT/chemo	in vivo	UM	[87]
**MSNPs**	functionalized MSNPs with photosensitizer and camptothecin	imaging/PDT/chemo	in vitro	RB	[90]
**polymer/lipid NPs**	β-Lap and photosensitizer in hybrid NPs doe	PDT/chemo	in vitro	RB	[91]
**SPIONS/liposomes**	superparamagnetic cationic nanoliposomes with ICG/PAI agent/PFH	imaging/PTT/PDT	in vivo	RB	[92]
**PLGA NPs**	PLGA nanoparticles with Ce6 and FeIII-TA	imaging/PTT/PDT	in vivo	UM	[93]
**AuNPs**	dosage enhancement of brachytherapy with 125I in the presence of AuNPs	Brachytherapy	Monte Carlo model	UM	[97]
**AuNPs**	dosage enhancement of brachytherapy with ^{103}Pd in the presence of AuNPs	Brachytherapy	Monte Carlo model	UM	[98]
**AuNPs**	comparative study evaluating ^{103}Pd and ^{125}I for NP-assisted brachytherapy	Brachytherapy	Monte Carlo model	UM	[99]
**AuNPs**	comparative study evaluating the parameters that affect dosimetry in NP-assisted brachytherapy	Brachytherapy	Monte Carlo model	UM	[100]
**AuNPs**	Brachytherapy of choroidal melanoma and lymphoma cells with AuNPs	Brachytherapy	in vitro	UM	[101]
**AuNPs**	distribution of AuNPs in an enucleated human eye with choroidal melanoma.	Brachytherapy	ex vivo	UM	[102]
**AuNPs**	Dosage distribution study in AuNP- assisted brachytherapy	Brachytherapy	Monte Carlo model	UM	[103]

**Iron Oxide NPs**	nanoparticle ferrofluid as shielding device in brachytrapy	Brachytherapy shied	Monte Carlo model	UM	[105]
**AuNPs**	^{125}I brachytherapy with AuNps and simultaneous ultrasonic hyperthermia	Brachytherapy/US hyperthermia	in vivo	RB	[106]
**lipid NPs**	Co-delivery of miR-181a and melphalan for seeded retinoblastoma	chemo/gene therapy	in vivo	RB	[123]
**lipid NPs**	imaging and laser-activated gene release	imaging/gene therapy	in vivo	RB	[124]
**PEI dendrimers**	study of gene transfection efficiency of RB cells with pDNA of RFP and GFP	gene therapy	in vitro	RB	[139]
**PEI dendrimers**	suicide gene therapy through transfection of OCM-1 cells with TNF-α/HSV-TK complex	gene therapy	in vitro	UM	[140]
**PEI dendrimers**	suicide gene therapy and radiotherapy of uveal melanoma OCM-1 cell line	radiotherapy/gene therapy	in vitro	UM	[141]
**PEI/AuNPs**	EpCAM-AuNP-PEI nanoconjugates to disrupt the EpCAM pathway in RB cells	gene therapy	in vitro	RB	[149]
**AuNPs**	detection and silencing of GNAQ mutations in UM cells	biomarker/gene therapy	in vitro	UM	[150]
**AuNPs**	AuNP-HDM2 to restore the functions of p53 in RB cells	gene therapy	in vitro	RB	[151]
**AuNPs/iron oxide**	magnetic gold nanocages with immunomodulator (MDP) and PFP for PA/US/MR imaging, low-intensity focused ultrasound (LIFU) therapy and immunotherapy	imaging/LIFU/immunotherapy	in vitro	RB	[152]
